# Supplementary material for: A randomized controlled trial testing a virtual program for Asian American women breast cancer survivors
Source: Nat Commun. 2023 Oct 14;14:6475. doi: 10.1038/s41467-023-42132-6 (PMC10576740; doi:10.1038/s41467-023-42132-6)
Supplement: Supplementary file 2 — Reporting Summary [file 41467_2023_42132_MOESM2_ESM.pdf]

nature portfolio

Corresponding author(s): Eun-Ok Im

Last updated by author(s): 07/17/2023

Reporting Summary

Nature Portfolio wishes to improve the reproducibility of the work that we publish. This form provides structure for consistency and transparency in reporting. For further information on Nature Portfolio policies, see our Editorial Policies and the Editorial Policy Checklist.

Statistics

For all statistical analyses, confirm that the following items are present in the figure legend, table legend, main text, or Methods section.

n/a	Confirmed	
☐	☒	The exact sample size (*n*) for each experimental group/condition, given as a discrete number and unit of measurement
☐	☒	A statement on whether measurements were taken from distinct samples or whether the same sample was measured repeatedly
☐	☒	The statistical test(s) used AND whether they are one- or two-sided<br>*Only common tests should be described solely by name; describe more complex techniques in the Methods section.*
☐	☒	A description of all covariates tested
☒	☐	A description of any assumptions or corrections, such as tests of normality and adjustment for multiple comparisons
☐	☒	A full description of the statistical parameters including central tendency (e.g. means) or other basic estimates (e.g. regression coefficient) AND variation (e.g. standard deviation) or associated estimates of uncertainty (e.g. confidence intervals)
☐	☒	For null hypothesis testing, the test statistic (e.g. *F*, *t*, *r*) with confidence intervals, effect sizes, degrees of freedom and *P* value noted<br>*Give P values as exact values whenever suitable.*
☒	☐	For Bayesian analysis, information on the choice of priors and Markov chain Monte Carlo settings
☒	☐	For hierarchical and complex designs, identification of the appropriate level for tests and full reporting of outcomes
☒	☐	Estimates of effect sizes (e.g. Cohen's *d*, Pearson's *r*), indicating how they were calculated

Our web collection on statistics for biologists contains articles on many of the points above.

Software and code

Policy information about availability of computer code

Data collection	The REDCap system was used for data collection.
Data analysis	The data from the REDCap were directly downloaded and used for the data analysis; Data analysis was performed using SPSS 28.0 software (IBM Corp., Armonk, NY, USA).

For manuscripts utilizing custom algorithms or software that are central to the research but not yet described in published literature, software must be made available to editors and reviewers. We strongly encourage code deposition in a community repository (e.g. GitHub). See the Nature Portfolio guidelines for submitting code & software for further information.

Data

Policy information about availability of data

All manuscripts must include a data availability statement. This statement should provide the following information, where applicable:

- Accession codes, unique identifiers, or web links for publicly available datasets
- A description of any restrictions on data availability
- For clinical datasets or third party data, please ensure that the statement adheres to our policy

Source data are provided with this paper. Yet, the data cannot be shared in a public repository because the research team did not get the permission from NIH or from the participants during the research process. However, any researchers could request the data sharing through contacting PI (Dr. Eun-Ok Im) and the data will be shared according to the following procedures that was approved by NIH at the time of the grant award. First, the data will be de-identified and the associated codebook that defines the data will be available for sharing with other researchers. The data will be available for secondary analyses especially by those who wish to

investigate the effectiveness of a culturally tailored technology-based information and coaching/support program in various variables other than our major outcome variables. Any researcher who wishes to use the data must request permission to conduct secondary analyses of the data from PI of the study (Dr. Eun-Ok Im) by e-mail or regular mail and provide PI with a 1-page long abstract (single-spaced) of the proposed analysis and his/her CV. The decision on data sharing will be made by the research team, including the PI, Co-Investigators, and consultants, after they review the abstract and CV. When the research team decides to share the data with the researcher, the data in SPSS format, abstract, and original findings will be provided to the researcher. The researcher will be requested to: (a) agree that she/he will provide the findings from her/his analyses to the PI at the completion of the analyses, (b) acknowledge the original study and the NIH in her/his future publications, and (c) not use the findings from the data for any commercial purposes. This agreement will be made in a written form. The data will have no identifying information to link a subject to her data. The data will be shared to the researcher through OneDrive. The data will be available for 10 years after the completion of the study.

Human research participants

Policy information about studies involving human research participants and Sex and Gender in Research.

Reporting on sex and gender	The gender of all participants was women. The gender of participants was determined based on the participants' self-reports. We only included self-reported Asian American women in this study because most of breast cancer survivors are women.
Population characteristics	Participants were self-reported Asian American women aged 21 years or older who had a breast cancer diagnosis, could read and write English, Mandarin Chinese, Korean or Japanese, and had access to the Internet.
Recruitment	The recruitment settings included both online and offline cancer support groups and communities/groups for Asian Americans. Those who visited the project website after reviewing the study announcements through the online and offline communities/groups were recruited (possible self-selection bias). Also, among over 71 sub-ethnic groups of Asian Americans, only three sub-ethnic groups (Chinese, Korean, and Japanese) were selected due to several reasons (e.g., language, East Asian cultural sphere), which could limit its generalizability to other subgroups. Also, this study required the participants to have regular access to the Internet, which might limit its generalizability to those with poor digital literacy or access. In addition, this study was limited to Asian Americans who identified as women and therefore did not include Asian American breast cancer survivors of other genders and gender identities.
Ethics oversight	The study was approved by the Institutional Review Board of Emory University.

Note that full information on the approval of the study protocol must also be provided in the manuscript.

Field-specific reporting

Please select the one below that is the best fit for your research. If you are not sure, read the appropriate sections before making your selection.

[x] Life sciences [ ] Behavioural & social sciences [ ] Ecological, evolutionary & environmental sciences

For a reference copy of the document with all sections, see nature.com/documents/nr-reporting-summary-flat.pdf

Life sciences study design

All studies must disclose on these points even when the disclosure is negative.

Sample size	In the sample size calculation, a sample size of 99 participants in each group (total N=198), with 3 repeated measurements obtained from each participant, would be adequately powered (80%) to detect a slope difference of 0.27, based on a two-group two-level hierarchical design. The calculation assumed a standard deviation of 1, a correlation of 0.1 between observations on the same subject, and an alpha level of 5%. The effect size was conventionally determined based on pilot studies.
Data exclusions	69 women were excluded during the data analysis process because they were not eligible because of several reasons (e.g., missing information on primary outcomes at the baseline).
Replication	TICAA (except the social media site) was independently and separately replicated for each participant in the intervention group (104 women).
Randomization	When volunteers met the inclusion criteria and quota requirements, only those who met the criteria and requirements were automatically given a serial number separately in each sub-ethnic group and randomized into two groups in each sub-ethnic group using an automated random number generator accessible through the website.
Blinding	After randomization, investigators, participants, and data analysts did not know which group the participants belonged to.

Reporting for specific materials, systems and methods

We require information from authors about some types of materials, experimental systems and methods used in many studies. Here, indicate whether each material, system or method listed is relevant to your study. If you are not sure if a list item applies to your research, read the appropriate section before selecting a response.

Materials & experimental systems

n/a	Involved in the study
☒	☐ Antibodies
☒	☐ Eukaryotic cell lines
☒	☐ Palaeontology and archaeology
☒	☐ Animals and other organisms
☐	☒ Clinical data
☒	☐ Dual use research of concern

Methods

n/a	Involved in the study
☒	☐ ChIP-seq
☒	☐ Flow cytometry
☒	☐ MRI-based neuroimaging

Clinical data

Policy information about clinical studies

All manuscripts should comply with the ICMJEguidelines for publication of clinical research and a completedCONSORT checklist must be included with all submissions.

Clinical trial registration	NCT02803593
Study protocol	Study protocol has been attached as a supplementary file.
Data collection	The recruitment settings included both online and offline cancer support groups and communities/groups for Asian Americans. Among over 71 sub-ethnic groups of Asian Americans, only three sub-ethnic groups (Chinese, Korean, and Japanese) were selected due to several reasons (e.g., language, East Asian cultural sphere). The data were collected from January 2017 to June 2020
Outcomes	The primary outcome was the needs score measured by the Support Care Needs Survey-34 Short Form (SCNS; primary). The secondary outcomes included the symptom distress score on the Memorial Symptom Assessment Scale-Short Form (MSAS) and the quality of life score on the Functional Assessment of Cancer Therapy Scale-Breast Cancer (FACT-B). In the clinicaltril.gov registration, all these outcomes are listed as primary outcomes, and mediators and moderators (that are not included in this paper) are listed as secondary outcomes.
