# Supplementary material for: A randomized controlled trial testing a virtual program for Asian American women breast cancer survivors
Source: Nat Commun. 2023 Oct 14;14:6475. doi: 10.1038/s41467-023-42132-6 (PMC10576740; doi:10.1038/s41467-023-42132-6)
Supplement: Supplementary file 3 — Description of Additional Supplementary Files [file 41467_2023_42132_MOESM3_ESM.pdf]

Description of Additional Supplementary Files

Supplementary Data 1
Description: Characteristics of the participants by group at the baseline (N= 199)
